# Supplementary material for: Effects of Dietary Supplementation with Honeybee Pollen and Its Supercritical Fluid Extract on Immune Response and Fillet’s Quality of Farmed Gilthead Seabream (Sparus aurata)
Source: Animals (Basel). 2022 Mar 8;12(6):675. doi: 10.3390/ani12060675 (PMC8944498; doi:10.3390/ani12060675)
Supplement: Supplementary file 1 [file animals-12-00675-s001.zip › animals-1552826-supplementary.pdf]

Table S1. Fatty acid profile (%) of honeybee pollen (HBP) and supercritical fluid extract (SFE) of HBP (HBP_SFE).

**Fatty acids**	**HBP**	**HBP_SFE**
C14:0	0.58±0.06	0.76±0.02
C16:0	24.82±0.06	24.51±0.20
C16:1n-7	0.17±0.00	0.19±0.00
C16:2n-4	1.59±0.21	2.56±0.05
C17:0	0.11±0.02	0.18±0.01
C16:3n-4	0.22±0.01	0.24±0.01
C18:0	2.02±0.08	2.20±0.08
C18:1n-9	5.72±0.41	6.93±0.17
C18:1n-7	0.74±0.01	0.68±0.02
C18:2n-6	16.54±0.36	14.26±0.24
C18:3n-3	42.86±1.20	43.53±0.48
C20:1n-9	1.15±0.12	1.53±0.03
C22:1n-11	1.83±0.19	2.44±0.05
C22:1n-9	1.66±0.19	n.d.
Σ SFA	27.53±0.10	27.64±0.09
Σ MUFA	11.27±0.53	11.77±0.28
Σ PUFA	61.21±0.63	60.59±0.18
Σ n-6PUFA	42.86±1.20	43.53±0.48
Σ n-3PUFA	16.54±0.35	14.26±0.24

n.d.: not detected.

Table S2. Growth performance indexes of gilthead se bream fed on diets added with 5% or 10% of raw HBP (diets P5 and P10) and 0.5% or 1% of HBP-SFE (diets E05 and E1).

	**CONTR**	**P5**	**P10**	**E0.5**	**E1**	***p*-Value**	**RMSE**
IBW	296.1	297.8	289.8	295.2	294.8	0.9709	14.80
FBW	343.6	347.6	318.0	335.3	336.7	0.2466	15.50
FCR	1.92	1.78	3.59	2.28	2.13	0.0807	0.74
SGR	0.47	0.48	0.29	0.40	0.42	0.1719	0.09
DIR‰	8.58	8.51	9.04	8.74	8.71	0.5600	0.40
WG%	16.16	16.74	9.74	13.67	14.25	0.1803	3.43
PER	1.26	1.32	0.75	1.06	1.11	0.1008	0.24

IBW = Initial Body Weight; FBW = Final Body Weight; Feed conversion ratio (FCR) = [total feed supplied (g)/weight gain (g)]; Specific growth rate (SGR, %/day) = [(lnFBW − lnIBW)/number of feeding days] * 100; Daily intake rate (DIR, ‰/day) = 1000 * [(feed intake (g)/mean weight (g))/days]; Weight gain (WG%) = 100 * [(FBW (g) – IBW (g))/IBW (g)]; Protein efficiency ratio (PER) = [weight gain (g)/total protein fed (g)].
